# Understanding Veterans’ Perceived Improvement in PTSD Treatment: Examining its Association with Clinical Predictors and Clinically Meaningful Improvement Thresholds

**DOI:** 10.1007/s10608-025-10637-7

**Published:** 2025-08-13

**Authors:** Emily E. Patton, Rhea Mundle, Sarah Pridgen, Philip Held

**Affiliations:** 1 Georgia State University, Atlanta, USA; 2 University of Mississippi, Oxford, USA; 3 Rush University Medical Center, Chicago, USA

## Abstract

**Background:**

Patients’ perceived improvement has utility in contextualizing markers of treatment success, often measured through PTSD severity, other clinical factors, and clinically meaningful improvement (CMI). This study investigated the relationship between perceived improvement, self-reported PTSD symptom changes, and changes in other clinical factors in veterans undergoing PTSD treatment.

**Methods:**

Data were collected from 259 veterans who completed a two-week Cognitive Processing Therapy-based intensive PTSD treatment program. Self-report measures, including the Patient Global Impression of Improvement (PGI-I) and PTSD Checklist for DSM-5 (PCL-5), assessed symptom severity and perceived improvement. Ordinal logistic regression analyses examined associations between PTSD symptom changes, other clinical factors (e.g., depression, self-efficacy, emotion regulation), and perceived improvement.

**Results:**

The average PCL-5 improvement was 20.93 points, with 87.65% of veterans reporting feeling at least a little better post-treatment. All CMI thresholds were related to perceived improvement. Reductions in PTSD severity significantly predicted higher perceived improvement while self-efficacy and emotion regulation also emerged as significant predictors. In contrast, changes in depression symptoms, negative posttraumatic cognitions, and resilience were not significant.

**Conclusions:**

Perceived improvement in PTSD treatment aligns closely with reductions in PTSD severity and self-regulatory capacities, emphasizing their importance in subjective recovery. While the PGI-I may not fully replace other measures, its alignment with key outcomes and brevity make it a valuable patient-centered tool for assessing treatment effectiveness. Future research should assess the PGI-I’s potential to complement or replace existing measures, and evaluate long-term outcomes across diverse treatment settings.

## Introduction

Patient-centered care emphasizes the importance of incorporating patients’ perspectives into treatment evaluation. One commonly used measure to assess patients’ perceived improvement in pharmacological treatments for psychiatric conditions is the Patient Global Impression of Improvement (PGI-I). The PGI-I asks individuals to rate their overall improvement compared to pre-treatment. The PGI-I provides a patient-centered perspective on treatment effectiveness, which can complement information from other symptom measures and aid in a comprehensive evaluation of treatment outcomes. Despite the PGI-I’s wide usage with other conditions, its application in the context of PTSD treatment is limited to medication studies (Pivac et al., 2004; Lindley et al., 2007). Further, there is limited research examining perceived improvement in PTSD treatment and the concordance between patients’ perceived improvement and CMI in individuals undergoing PTSD treatment. Incorporating these perspectives not only complements symptom-based measures but also enhances the understanding of efficacy and patient recovery. Exploring perceived improvement offers a unique opportunity to assess recovery through the lens of patients’ subjective experiences. Understanding how self-reported symptom improvements compare to patients’ perceived improvements may be more patient-centered and help improve existing ways of evaluating meaningful change and treatment response.

Clinically meaningful improvement (CMI) is a widely used benchmark to assess efficacy in both treatment trials and in clinical practice (Bisson et al., 2013). CMI is operationalized as when symptom severity reduction is greater than what can be attributed to chance and is associated with improved functioning (Jacobson et al., 1984, 1999; Kazdin, 1999). Determining whether patients have reached CMI can help inform treatment planning, such as determining when to discontinue treatment, depending on collaboratively agreed upon therapeutic goals (Lambert et al., 2001).

In PTSD treatment, the widespread use of standardized self-reported symptom measures, such as the PTSD Checklist for DSM-5 (PCL-5; Weathers et al., 2013), has allowed for the quantification of symptom severity and performance monitoring in routine care. CMI on the PCL-5 is frequently used to demarcate PTSD treatment success (Benfer et al., 2023), although there is no single agreed upon definition. Changes of 10–15 points on the PCL-5 are commonly considered CMI (Marx et al., 2022; U.S. Department of Veterans Affairs, n.d.). In addition to CMI, different studies have also used the PCL-5 to estimate whether patients are likely to no longer meet the diagnostic threshold for PTSD, which is another important indicator of treatment success. Depending on the study, PCL-5 scores above 22, 28, 31, and 33 have been suggested to be indicative of meeting the diagnostic criteria for PTSD (Marx et al., 2022; U.S. Department of Veterans Affairs, n.d.; Weathers et al., 2013; Mathersul et al., 2019; Blevins et al., 2015; Bovin et al., 2016; Benfer et al., 2023).

CMI thresholds are valuable for standardizing outcome assessment and demonstrating treatment efficacy. However, they also have several notable limitations. For example, although CMI describes symptom change that is greater than what could be expected due to chance, to a patient these arbitrary cutoffs may not fully capture their lived experiences of improvement. Another challenge associated with using CMI is the lack of agreed upon thresholds for the same measure. Although PCL-5 change scores of 10- or 15-points are most commonly used to indicate CMI (Benfer et al., 2023; Marx et al., 2022; U.S. Department of Veterans Affairs, n.d.), thresholds such as 12- (Steenkamp et al., 2015) or 18-point PCL-5 change (Marx et al., 2022) have also been suggested. Most of these thresholds were established by comparing self-reported symptom changes to clinician-rated symptom change and did not take patient perceptions into account. Additionally, the assessment burden poses a significant challenge, as many individuals are fatigued by repeatedly completing lengthy measures (Rolstad et al., 2011). Implementing a single-item measure that is equally effective at tracking improvement would greatly alleviate this burden and enhance the feasibility of ongoing assessments (Bize & Plotnikoff, 2009; Hoeppner et al., 2011). As these thresholds examine PTSD symptom reduction alone, they may overlook improvements that are meaningful to patients, underscoring the need for complementary measures like the PGI-I.

Few studies to date have determined the extent to which each threshold is associated with patients’ perceived improvements in well-being or functioning. Further, not many studies have examined how perceived improvement is associated with symptom change more broadly. In a treatment study with veterans with current PTSD and substance use (*N* = 52), perceived improvement persisted and increased throughout treatment (Najavits et al., 2018). This finding aligns with research conducted in other conditions where perceived improvement was positively associated with symptom reductions in emotional stability (Andrade et al., 2012) and mood symptoms (Andrade et al., 2012; Potes et al., 2016). Previous research by Matthews et al. (2022) revealed a positive association between perceived improvement and established PTSD clinical thresholds (31-point PCL-5 cut-off indicating benefit from treatment, 19-point PCL-5 cut-off indicating recovery). Individuals who fell below these thresholds tended to also perceive greater improvement. However, when patients were asked what change they thought they would require in order to notice feeling better, between 15 and 21% of patients required significantly greater symptom improvement than the clinical thresholds commonly used in clinical research and practice (31- and 19-point cut-off; Matthews et al., 2022).

Although perceived improvement may be influenced by PTSD, other factors could also impact patients’ ability to notice improvements. Kazdin argued that clinical significance is not restricted to changes in symptoms alone but may also incorporate other constructs and highlights the importance of assessing patients’ perceived improvement (1999). Notably, research has demonstrated that improvements in self-reported PTSD severity are associated with improvements in a variety of domains, including functioning, social and occupational impairment, and emotion regulation (Schnurr & Lunney, 2016; Lunney & Schnurr, 2007). Further, previous research has highlighted the association between emotion regulation, posttraumatic cognitions, and resilience, functioning, and perceived well-being (Cherry & Wilcox, 2020; Chow et al., 2018; Liu et al., 2015; Sarrionandia et al., 2018; Wells et al., 2019). Following Kazdin’s (1999) logic, metrics like PGI-I would potentially capture improvements in all of these. However, it is currently unknown to what extent each of these factors may be driving patients’ perceived improvement when assessed together with PTSD severity.

This study examined the relationship between perceived improvement, PTSD symptom change, and change in other important clinical factors in veterans undergoing PTSD treatment. First, we evaluated how previously established CMI cutoffs for self-reported PTSD symptoms (10- and 15-point PCL-5 change), as well as meeting the common probable PTSD diagnostic cutoff score of 33 points on the PCL-5 related to veterans’ perceived symptom improvement at the end of treatment. Next, we added changes in additional symptom- and functioning-based clinical factors (i.e., depression, self-efficacy, resilience, emotion regulation, posttraumatic negative cognitions) to the predictive model that previously only included PTSD to determine the extent to which each of these constructs related to patients’ perceived improvement. By exploring these relationships, this study aims to provide insights into how perceived improvement correlates with a range of clinical factors and clinical improvement after treatment. Further, it could determine the extent to which a single item assessing perceived improvement is able to capture improvement reported on various validated measures, which has important implications for reducing assessment burden, especially in clinics where specific measures are not used for research purposes.

## Methods

### Participants

Data were collected from 259 veterans who completed an in-person 2-week Cognitive Processing Therapy (CPT)-based intensive PTSD treatment program (ITP) (Held, 2020). The veterans in the present sample were on average 43.56 years old (*SD* = 10.02) and identified as primarily white (64.86%) and male (54.83%; see Table 1 for additional demographic characteristics). All study procedures were approved by the Rush University Medical Center Institutional Review Board (Held, 2020), and all participants provided informed consent prior to their participation in the study. All procedures were conducted in accordance with the ethical standards outlined in the Belmont Report.

Veterans who presented with issues that might interfere with their active participation in the ITP, such as unstable housing or serious medical illness, were not eligible for the program. Further ineligibility criteria included a need for a higher level of care due to active suicidality or homicidality, unmanaged mania or psychosis, or substance use with the potential for hospitalization if discontinued abruptly.

### Intensive Treatment Program for PTSD

Cognitive Processing Therapy is a trauma-focused first line evidence-based treatment for PTSD that has been demonstrated to be effective (See Resick et al., 2024 for more information). While CPT has traditionally been conducted over 12 weekly sessions, intensive formats of CPT with daily sessions have been shown to be effective and advantageous in reducing length of treatment (Held et al.,2024; Held et al., 2023a, b). During the 2-week ITP, veterans received twice daily individual CPT sessions (16 total, 50-minutes each). Each session was followed by a 60-minute block to complete homework assignments, several 50-minute psycho-educational groups (e.g., sleep hygiene, cognitive health), and adjunctive services (e.g., art therapy, acupuncture; more information about the program is available in Authors et al., 2023).

### Procedures

The intake process for veterans seeking treatment in the ITP involved a combination of clinician-administered assessments and self-report measures to evaluate PTSD, along with other mental and physical health symptoms. To qualify, veterans had to meet the diagnostic criteria for PTSD as determined by the Clinician-Administered Scale for PTSD for DSM-5 (CAPS-5; Weathers et al., 2013).

### Measures

#### Demographic Measures

At baseline, veterans reported demographic and military service-related information (i.e., race, gender, ethnicity).

#### Patient Global Impression Scale of Improvement (PGI-I; Hossack & Woo, *2014;* Steinert et al., 2010)

The PGI-I is a single item self-report measure to assess patients’ interpretation of symptom improvement following treatment. Veterans rated how much they believed to have improved compared to before treatment on a scale of 1 = very much better to 7 = very much worse, with lower scores indicating higher improvement. This measure was administered at post-treatment. In previous research, the PGI-I has been found to be associated with depression and bipolar disorder symptoms (Mohebbi et al., 2018; Demyttenaere et al., 2021).

#### PTSD Checklist for DSM-5 (PCL-5; Weathers et al., 2013)

The PCL-5 is a 20-item self-report measure that assesses PTSD symptom severity based on DSM-5 diagnostic criteria. Veterans rated their symptoms as related to the index trauma targeted in treatment. Items were rated on a scale from 0 = not at all to 4 = extremely. Higher scores indicate higher PTSD symptom severity with total scores ranging from 0 to 80. PCL-5 was administered at both baseline asking about past month symptoms and post-treatment asking about past week symptoms. Cronbach’s alpha for this sample ranged from 0.92 to 0.96.

#### Patient Health Questionnaire (PHQ-9; Kroenke et al., 2001)

The PHQ-9 is a 9-item self-report measure for depressive symptoms in the past two weeks. Each item was rated from 0 = not at all to 3 = nearly every day. Total score for the PHQ-9 ranges from 0 to 27, with higher scores indicating higher depressive symptom severity. This measure was administered at both baseline and post-treatment. The internal reliability for this sample ranged from 0.84 to 0.88.

#### Posttraumatic Cognitions Inventory (PTCI; Foa et al., 1999)

The PTCI is a 33-item self-report questionnaire that measures trauma related thoughts and beliefs. Items were rated from 1 = totally disagree to 7 = totally agree. Total scores range from 33 to 231, with higher scores indicating more strongly held negative trauma-related beliefs. This measure was administered at baseline and post-treatment. The internal reliability for this sample ranged from 0.95 to 0.98.

#### Self-Efficacy for Managing Chronic Conditions-Managing Symptoms v1.0- Short Form 8a (PROMIS SE; Gruber-Baldini et al., 2017)

The PROMIS SE is an 8-item self-report measure assessing self-efficacy in 5 domains. Three items related to managing medication and treatment were not included^1^. Our study measured only 5 items of self-efficacy for managing daily activities, symptoms, emotions, and social interactions. Veterans rated each item from 1 = not at all confident to 5 = very confident, with higher scores indicating higher self-efficacy. This measure was assessed at baseline and post-treatment. The internal reliability for this sample ranged from 0.80 to 0.93.

#### Connor-Davidson Resilience Scale 10 (CD-RISC-10; Campbell-Sills & Stein, 2007)

The CD-RISC-10 is a 10-item self-report measure of resilience in the past month. Resilience is captured in this measure by assessing one’s ability to cope with adversity. Specifically, veterans were asked to respond to items about their abilities to adapt to change, see the humorous side of problems, achieve goals despite obstacles, handle unpleasant feelings, etc. Veterans rated items on a 5-point Likert scale from 0 = not true at all to 4 = true nearly all the time. Higher scores indicate greater resilience. The CD-RISC-10 was administered at baseline and post-treatment. The internal reliability for this sample ranged from 0.88 to 0.91.

#### Difficulties in Emotion Regulation Scale- Short Form (DERS-SF; Kaufman et al., 2016)

The DERS-SF is an 18-item self-report measure that assesses issues in emotion regulation with subscales for strategies, non-acceptance, impulse, goals, awareness, and clarity. The items are each rated from almost never (0–10%) to almost always (91–100%) with higher scores indicating greater difficulty in emotion regulation. This measure was administered at baseline and post-treatment. The internal reliability for this sample ranged from 0.89 to 0.93.

### Statistical Analysis

All analyses, including descriptive statistics that were calculated to summarize the demographic and clinical thresholds of the study sample, including means, standard deviations, frequencies, and percentages were conducted using R version 4.3.0 (R Core Team, 2022). To examine whether changes in PCL-5 predicted post-treatment PGI-I (1 = very much better to 7 = very much worse), we used an ordinal logistic regression. Next, two variables were dummy coded for the 10- and 15-point thresholds to indicate whether change in PCL-5 from pre- to post-treatment was greater than or equal to common CMI thresholds or not (1 = met clinically meaningful change indicators, 0 = did not meet clinically meaningful change indicators). A third variable was dummy coded for the probable diagnostic cut-off (< 33 PCL-5 total score) as post-treatment (1 = did not meet for probable PTSD, 0 = met for probable PTSD). Separate ordinal logistic regressions were conducted to examine the degree to which each threshold predicted post-treatment PGI-I.

To examine the relationship between the clinical factors (e.g., PCL-5, PHQ-9, PTCI, PROMIS SE, CD-RISC-10, DERS-SF) and post-treatment PGI-I, an ordinal logistic regression was conducted controlling for sex and race. These were included due to the sex differences related to treatment identified in previous literature (Rutt et al., 2018) and contradictory treatment outcomes differences identified with race (McClendon et al., 2020). Prior to conducting the regression analysis, multicollinearity among the predictor variables was assessed using variance inflation factors (VIFs). Using a conservative threshold, variables with VIF values exceeding 3 were considered indicative of collinearity issues as it suggests a potential suppression of variables and these variables were further examined for potential removal from the model (Menard, 2002). Assumptions for ordinal logistic regression, including normality, homosce-dasticity, and absence of multicollinearity were assessed and met, suggesting that analyses were appropriate. As ordinal logistic regression output does not include R-squared values, McFadden’s pseudo R-squared was calculated for each model. McFadden’s pseudo R-squared was selected as it considers R-squared as improvement from null model to fitted model and views R-squared as explained variability (Long, 1997).

## Results

Overall, the sample showed an average PCL-5 improvement of 20.93 points (SD = 16.32, d = 1.28), and 87.65% of veterans reported feeling at least a little better compared to when they started treatment on the PGI-I (M = 2.55, SD = 1.31; *V* = 27160, *p* < 0.05). In our sample of 259 veterans, 189 (73%) made at least a 10-point PCL-5 reduction, 161 (62%) made at least a 15-point reduction, and 128 (49%) fell below the suggested threshold for probable PTSD (i.e., < 33 PCL-5 points) by the end of treatment. Among participants, 220 (84.94%) reported perceived improvement that aligned with whether they reached CMI. Most (*n* = 196, 75.68%) reported that they felt better and met at least one CMI criterion. A smaller subset of 39 veterans (15.06%) reported perceived improvement that did not align with whether they reached CMI, with the majority of these participants (*n* = 31, 11.97%) indicating that they did not feel better despite meeting at least one CMI criterion.

Greater pre- to post-treatment reductions in PTSD severity were significantly associated with higher perceived improvement at post-treatment (*b* =−0.091, *p* < 0.001 *pseudo R^2^* = 0.16; see Table 2). Further analyses revealed that meeting CMI criteria was significantly associated with perceived improvement, regardless of the specific PCL-5 clinical indicator. Specifically, veterans achieving at least a 10-point PCL-5 reduction had 11.35 times higher odds of reporting greater perceived improvement (OR = 11.35, *p* < 0.001, *pseudo R^2^* = 0.098). Similarly, those with at least a 15-point PCL-5 reduction had 14.73 times higher odds of reporting greater perceived improvement (OR = 14.73, *p* < 0.001, *pseudo R^2^* = 0.126). Falling below the probable PTSD threshold (PCL-5 < 33) was also strongly associated with perceived improvement, with veterans in this category having 18.89 times higher odds of reporting better perceived improvement (OR = 18.89, *p* < 0.001 *pseudo R^2^* = 0.148).

Including all clinical predictors, the full model explained 42.93% of the variance in perceived improvement. Pre- to post-treatment reductions in PTSD severity (*b* = − 0.027, *p* < 0.001, self-efficacy (*b* = 0.060, *p* < 0.001, and improvements in emotion regulation (*b* = − 0.015, *p* = 0.013) were significantly associated with perceived improvement (see Table 3). In contrast, depression (*b* = 0.007, *p* = 0.646), resilience (*b* = 0.013, *p* = 0.160), negative posttraumatic cognitions (*b* = − 0.001, *p* = 0.777), race (*b* = − 0.002, *p* = 0.993), and sex (*b* = 0.052, *p* = 0.643) were not significantly associated with perceived improvement.

## Discussion

To our knowledge, this study was the first to examine how previously established definitions of clinically meaningful improvement (CMI) and suggested probable diagnostic threshold definitions based on self-report PTSD scales relate to perceived improvements reported by veterans undergoing PTSD treatment. Previous research has identified significant associations between perceived improvement and reductions in distress across PTSD and other conditions (Andrade et al., 2012; Matthews et al., 2022; Najavits et al., 2018; Potes et al., 2016), supporting our findings that perceived improvement significantly aligns with changes in PTSD symptom severity. Of the 259 participants, 75.68% reported perceived improvement and met at least one of the established CMI thresholds. Among only those who reported perceived improvement (*n* = 227), 84.94% met a CMI threshold.

Importantly, this study found that the CMI and suggested probable diagnostic thresholds explained similar amounts of variance in perceived improvement, albeit minimal (no more than 15%). The relatively small differences in the amount of variance across the 33-point cut-off, 10-point, and 15-point PCL-5 change thresholds suggest that all options reasonably reflect perceived improvement, similar to what was found by Matthes and colleagues (2022). When examining odds ratios those who fell below the 33-point probable PTSD cutoff had 18.89 times higher odds of perceiving improvement. This means that meeting the 33-point probable PTSD cutoff is associated with 66% greater odds of perceived improvement than meeting the 10-point threshold and 28% greater than meeting the 15-point threshold. These findings underscore that while all thresholds are meaningful, the 33-point probable PTSD cutoff may be the strongest predictor of perceived improvement. Nevertheless, the modest explanatory power highlights the potential need for additional factors to fully capture patients’ subjective experiences of improvement, aligning with past studies that have emphasized the multidimensionality of perceived improvement (Kazdin, 1999).

Clinicians and researchers may find that incorporating patient-centered measures like the PGI-I provides additional context to traditional symptom-based assessments. While PGI-I demonstrated strong alignment with PTSD symptom reduction, self-efficacy, and emotion regulation, it does not fully capture symptom-level changes or all facets of treatment response. Therefore, we do not recommend replacing existing measures with PGI-I at this stage. Instead, PGI-I may serve as a useful complementary measure, helping clinicians gauge whether patients feel meaningfully better even if they have not met a specific symptom reduction threshold. Future research should explore whether PGI-I could be used as a standalone measure in certain contexts, such as initial treatment response monitoring or patient-driven care models.

It has been suggested that impairments in meeting role demands, interactions with others, and ability restrictions may be regarded as much more critical than mental health symptoms for someone seeking treatment (Kazdin, 1999). Thus, we reasoned that it was important to examine what may impact perceived improvement as PTSD severity change alone did not completely explain the variance. Overall, our findings highlight the multifaceted nature of perceived improvement within the context of PTSD treatment. We found that improvements in PTSD severity remained significant even when taking into account other clinical factors; however this construct alone did not explain the variance entirely. Self-efficacy and emotion regulation also contributed, underscoring their potential role in subjective improvement. These findings align with previous studies demonstrating that improvements in self-reported PTSD severity are consistently associated with positive changes in functioning and emotion regulation (Hamrick et al., 2023; Hinton et al., 2021; Schnurr & Lunney, 2016; Lunney & Schnurr, 2007).

Moreover, studies of veterans undergoing PTSD treatment (Najavits et al., 2018) have noted an improvement in perceived symptom improvement after treatment, highlighting the strong association between the severity of PTSD and patients’ subjective assessments of their progress. The association of additional factors such as self-efficacy with perceived improvement may be explained by their combined impact on an individual’s overall well-being and their influence on patients’ perceived ability to manage their symptoms. In other words, feeling more capable of managing one’s PTSD symptoms likely represents an important marker of improvement, especially as this is the main reason individuals seek PTSD treatment in the first place. This aligns with previous research which demonstrated that quality of life-oriented factors are associated with decreases in PTSD symptom severity (Schnurr & Lunney, 2016; Lunney & Schnurr, 2007). Recognizing that individuals feel more capable of managing PTSD symptoms may represent an important marker of recovery, particularly as this capability often underpins patients’ goals for seeking treatment. Testing this process goes beyond this manuscript, is purely speculative, and should be examined in future research to better understand how the identified factors may contribute to perceived improvement.

Despite our expectations, changes in depression symptoms, resilience, and negative posttraumatic cognitions were not significant predictors of perceived improvements at the end of treatment when entered into the same model as PTSD symptoms and the aforementioned predictors. A possible explanation may be their substantial overlap with other constructs, such as PTSD severity (Brown et al., 2019; Stander et al., 2014), which measure sleep (e.g., PHQ-9) or changes in cognitions (e.g., PTCI) which are also part of PTSD. Alternatively, these measures may not fully capture the nuanced, subjective experiences that inform perceived improvement. In other words, some individuals may perceive changes in their beliefs even without a corresponding improvement in symptoms (Held et al., 2023a, b).

This study has several limitations that should be taken into consideration. First, the groupings of individuals into CMI categories overlap to some extent (e.g., all those in the 10-point PCL-5 threshold group are also automatically in the 15-point PCL-5 threshold group). This overlap could introduce potential for ambiguity in determining treatment response and perceived improvement. Second, the variables included in the model for examining factors related to perceived improvement were limited and selected based on availability. Although the selected function-based variables (e.g., self-efficacy, emotion regulation) were theoretically relevant based on the limited literature, there may have been other constructs unavailable in this sample that contribute to perceived improvement such as quality of life, treatment satisfaction, or activities of daily living. Future studies should consider incorporating a broader range of factors to provide a more comprehensive understanding of the complex nature of perceived improvement. Based on these findings and previous literature, function-based factors seem especially important to examine. Additionally, many of the measures were self-reported and asked about the past week or two at treatment endpoint and, thus, may not reflect patients’ true state at endpoint.

Despite these limitations, this study contributes valuable insights into factors associated with perceived improvement in PTSD treatment. Future research should include longer follow-up periods to assess whether perceived improvement at the end of treatment persists over time and examine any variance in factors that impact those perceptions. Future research should attempt to replicate these findings in other samples or treatment settings to identify factors that are consistently associated with perceived improvement. It could also be valuable to consider prioritizing perceived improvement as an additional primary outcome in addition to assessing symptom severity. By focusing on individuals’ subjective experiences and their perception of improvement, treatment interventions can be tailored to address not only symptom severity but also personal evaluations of progress. Incorporating patients’ perspectives and treatment goals into collaborative decision-making processes fosters more personalized and patient-centered care, ultimately enhancing treatment outcomes, increasing patient satisfaction, and potentially reducing treatment dropout. Although the single-item measure tested in the current study did not capture the full range of variance in patient-reported outcomes, it may still offer utility in monitoring general progress. Continued research is needed to explore whether shorter assessments, including single-item measures, can meaningfully reduce assessment burden while maintaining clinical utility.

## Figures and Tables

**Table 1:** Demographic characteristics of sample (N = 259)

Characteristic	*n*	%
*Sex*		
Male	142	54.83
Female	117	45.17
Ethnicity		
Not Hispanic	217	83.78
*Race*		
Native American/Alaskan Native	1	0.39
Asian	8	3.09
Black or African American	63	24.32
Native Hawaiian or Pacific Islander	4	1.54
White	168	64.86
Other	15	5.79
*Military Service Branch*		
Air Force	35	13.51
Army	130	50.19
Coast Guard	20	7.72
Marines	36	13.90
Navy	38	14.67
*Service Era*		
After September 11, 2001	207	79.92
*Deployed*		
Yes	179	69.11

**Table 2:** Models of PTSD improvement criteria predicting perceived improvement

Models	b	SE	*p*	Pseudo *R*^2^	OR
*Model 1*: 10-point PCL-5 Change	−2.44	0.31	< 0.001	0.10	11.35
*Model 2*: 15-point PCL-5 Change	−2.59	0.30	< 0.001	0.13	14.73
*Model 3*: 33-point Probable PTSD Cut-off	−2.74	0.30	< 0.001	0.15	18.89

**Table 3:** Predictors of perceived improvement

Variables	b	SE	*p*
Δ PCL-5^a^	−0.026	0.005	< 0.001
Δ PHQ-9^b^	0.007	0.015	0.646
Δ CD-RISC-10^c^	0.013	0.009	0.160
Δ PROMIS SE^d^	0.060	0.015	< 0.001
Δ DERS-SF^e^	−0.015	0.006	0.013
Δ PTCI^f^	−0.001	0.002	0.777
Race	−0.002	0.118	0.983
Sex	0.052	0.113	0.644

**Note** Perceived improvement was measured via the Patient Global Impression scale of Improvement (PGI-I) at post-treatment. Perceived improvement was measured via the Patient Global Impression scale of Improvement (PGI-I) at post-treatment. Predictors were based off pre- to post-treatment change.

a PTSD Severity,

b Depression,

c Resilience,

d Self-efficacy,

e Emotion regulation difficulties,

f Posttraumatic Cognitions Inventory.

## Data Availability

The datasets used in the current study are not publicly available. Datasets can be obtained from the corresponding author upon reasonable request.

## Footnotes

1 Items “*I can work with my doctor to manage my symptoms*” and “*I can find the information I need to manage my symptoms*” were removed from the survey as they were not related to the IOP. “*I can keep my symptoms from interfering with my personal care*” was also removed as this information was better captured elsewhere.
